# Papillary Thyroid Microcarcinoma with a Large Cystic Dilated Lymph Node Metastasis to the Neck Mimicking a Branchial Cleft Cyst: A Potential Pitfall

**DOI:** 10.1155/2015/796358

**Published:** 2015-07-09

**Authors:** Osman Ilkay Ozdamar, Gul Ozbilen Acar, Cigdem Kafkasli, M. Tayyar Kalcioglu, Tulay Zenginkinet, H. Gonca Tamer

**Affiliations:** ^1^Department of Otorhinolaryngology-Head and Neck Surgery, Istanbul Medeniyet University Goztepe Training and Research Hospital, 34730 Istanbul, Turkey; ^2^Department of Pathology, Istanbul Medeniyet University Goztepe Training and Research Hospital, 34730 Istanbul, Turkey; ^3^Department of Endocrinology and Metabolic Diseases, Istanbul Medeniyet University Goztepe Training and Research Hospital, 34730 Istanbul, Turkey

## Abstract

Lateral cervical cystic mass in a young adult very rarely could be a first sign of an occult thyroid papillary microcarcinoma metastasis. In this paper, we presented a 37-year-old male patient whose preoperative 6 cm left lateral cervical cystic mass was initially diagnosed as branchial cleft cyst, but then the postoperative histopathological examination of the mass was revealed as papillary thyroid carcinoma metastasis. Preoperative fine needle aspiration biopsy was relevant with a branchial cleft cyst. In the left thyroid lobe there were 3 solid nodules with 4, 6, and 12 mm dimensions, respectively. One of the nodules had malignant well-differentiated cells diagnosed after fine needle aspiration biopsy. After total thyroidectomy, histopathologic evaluation of biopsy material's showed papillary thyroid microcarcinomas. This case indicates that especially in a young adult lateral cervical cystic mass should be carefully considered preoperatively for the possibility of metastatic occult thyroid carcinoma, especially for papillary carcinoma in differential diagnosis, and evaluation of the thyroid gland should be taken into account.

## 1. Introduction

Branchial cleft cyst is a congenital malformation that resulted from various degrees of incomplete closure of branchial sinus during embryogenesis. Although it is a congenital malformation, its clinical presentation is usually through the second to fourth decades of life that is mostly following an infection of the cyst.

Papillary thyroid carcinoma is the most common and well-differentiated malignant tumor of the thyroid gland. Tumor size that is equal to or smaller than 10 mm in diameter is labeled as thyroid papillary microcarcinoma according to World Health Organization (WHO) [1]. Cervical cystic neck mass in a young adult, which is located classically anterior to the sternocleidomastoid muscle (SCM), is most commonly branchial cleft cyst if otherwise proven. Lateral cervical cystic mass in a young adult very rarely could be a first sign of an occult thyroid papillary microcarcinoma metastasis.

A lateral cervical cystic lesion mimicking a branchial cyst can harbor and hide a well-differentiated thyroid malignant tumor metastasis, which is disclosed only after the histopathological examination of the specimen that is definitely making the correct diagnosis possible. This rare phenomenon has been mentioned in the literature [2–4].

In this paper, we discuss a case with a large lateral cervical cyst that was initially diagnosed as branchial cleft cyst but after histopathological examination it was diagnosed to be a cystic enlarged lymph node which was also invaded with thyroid papillary carcinoma, with the review of the literature.

## 2. Case

A 37-year-old male patient applied to our clinic with a complaint of a neck mass that had enlarged approximately within 3 months on the left lateral side of the neck. He had no additional complaint. The mass was semimobile, smooth in consistency, and painless which was located anterior to the left sternocleidomastoid (SCM) muscle. There was no other palpable mass on examination of the neck. His medical and family histories were unremarkable. Other otolaryngologic and endoscopic examinations were normal. Fine needle aspiration biopsy (FNAB) of the mass was doubtful with no malignant cells. There was no palpable mass on the neck of the patient, except only the lateral cervical cystic mass.

**Figure 1:**
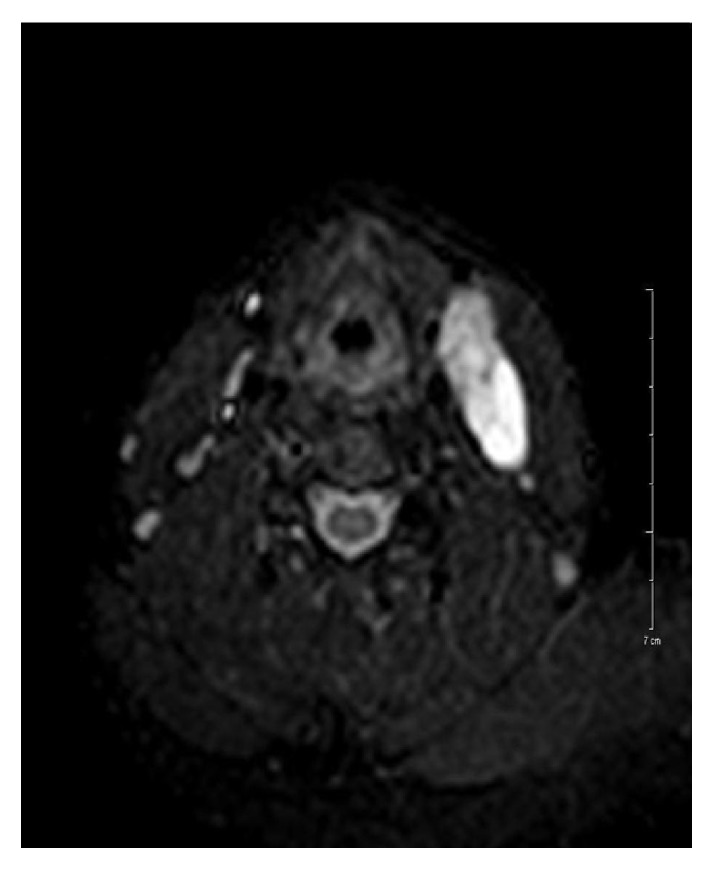
Contrast enhanced, axial sliced MR imaging of the cystic lesion.

**Figure 2:**
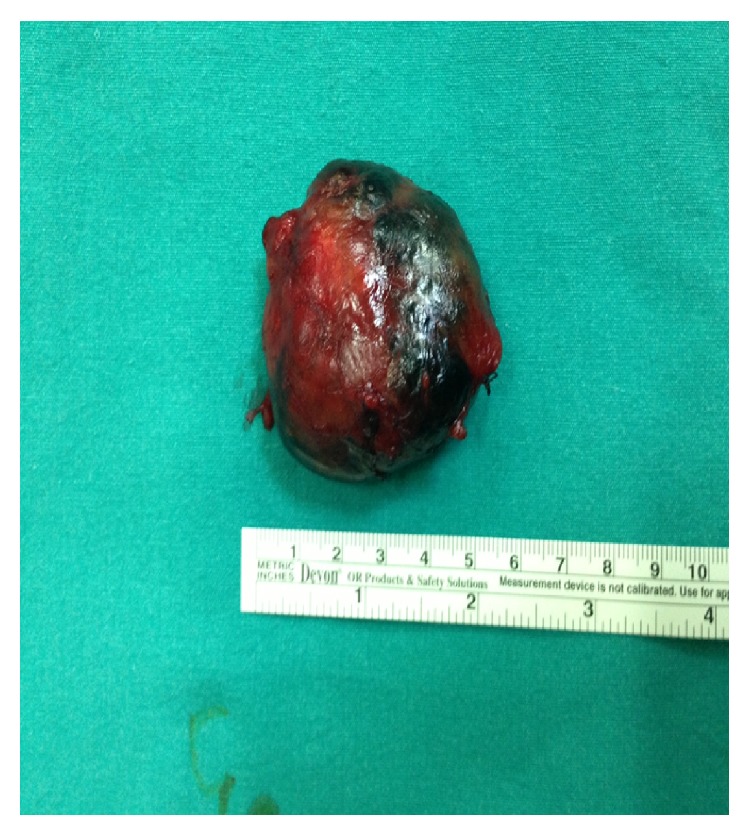
Postoperative appearance of the cystic mass after total excision.

Nearly 6 cm cystic mass that was filled with fluid was detected on contrast enhanced magnetic resonance imaging (MRI) (Figure 1). Near the cyst approximately 1 cm in diameter 2 lymph nodes that were relevant with reactive nodes were also detected. An approximately 6 cm large cystic mass on the left lateral side on the neck was extirpated (Figure 2).

**Figure 3:**
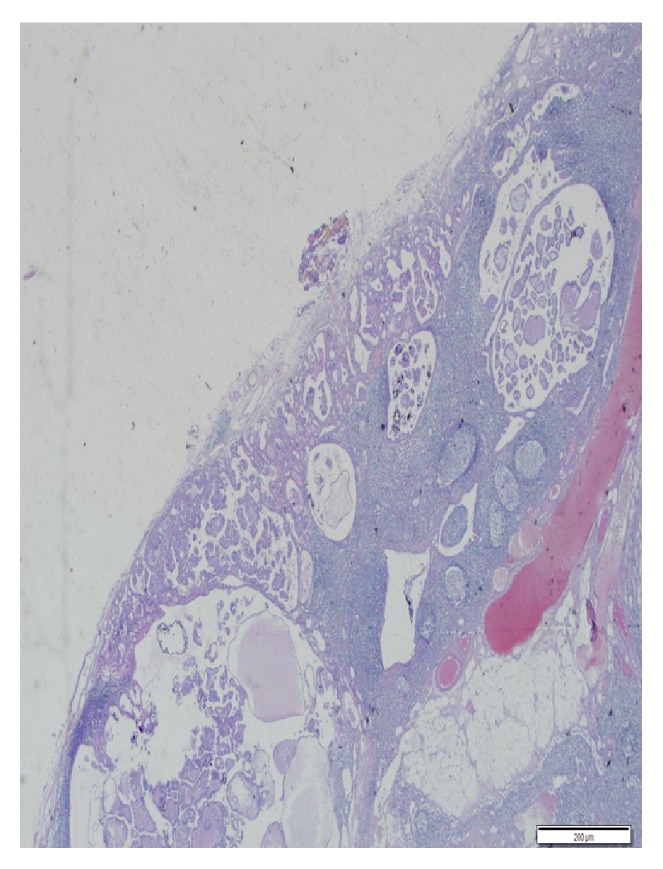
Metastatic focus of papillary carcinoma. It forms cystic papillary structures that are advancing from subcapsular region to the center of the cystic lymph node (H&E ×100).

**Figure 4:**
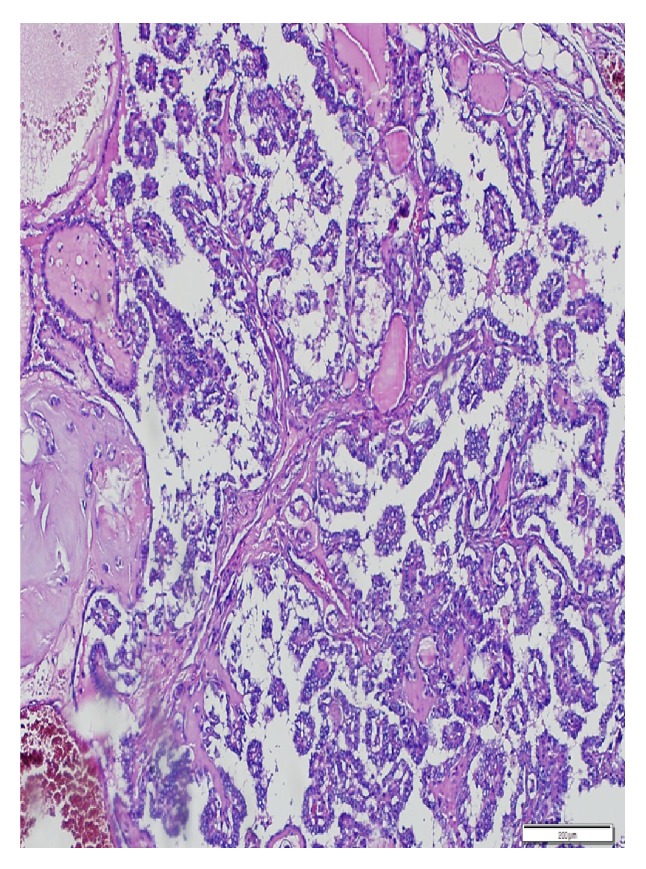
Papillary structures are laid down by laminated cells with clear nucleus (H&E ×100).

Cervical cystic mass was initially diagnosed as branchial cleft cyst, but then the postoperative histopathological examination of the mass was revealed as papillary thyroid carcinoma metastasis (Figure 3). Thyroid function tests were normal. Ultrasonographic (USG) examination of the thyroid gland showed that the left lobe of thyroid gland had 3 solid nodules with 4, 6, and 12 mm in largest diameter, respectively, but no nodule was found in the right lobe and also in the isthmus of the thyroid gland. FNA biopsies were performed to suspected nodules, but no malignant cell was detected except in one 6 mm nodule, which was suspected having malignant well-differentiated cells. Contrast enhanced computerized tomography (CT) was also performed to disclose mediastinal lymph node metastasis if it exists. Total thyroidectomy was performed to the patient, and postoperative histopathological examination of biopsy material's result showed two papillary thyroid carcinomas with dimensions of 4 and 6 mm, respectively (Figure 4). Nevertheless, 12 mm sized dominant nodule had no malignancy. The patient underwent a radioactive iodine (RAI) ablation therapy 7 weeks after the operation, with planning of thyroid hormone replacement therapy thereafter. Laboratory measurement values of TSH and thyroglobulin with laboratory reference values just before radioactive ablation therapy were 68.90 *μ*IU/mL (0.27–4.2) and 0.72 ng/mL (1.6–59.9), respectively. The patient has been free of disease for more than 1 year.

## 3. Discussion 

Branchial cleft cyst is a common congenital developmental abnormality in young adults. Its most common presentation is second branchial cleft cyst, which is located classically anterior to the SCM [5]. Although cystic neck masses are mostly benign lesions in young adults, rarely, it may be the first and sole clinical symptom of a well-differentiated, almost all papillary, thyroid carcinoma [2, 3].

To minimize imaging artifacts, MRI was performed before the USG guided FNAB. MRI and FNAB results were compatible with branchial cyst with its classical anatomic location, so we decided as an overdiagnostic tool to perform a neck CT and secondarily to avoid extra radiation dose to the patient. It is concluded in the literature that thyroid nodules have missed despite to performed neck CT and/or MRI as in our case, but the reason of that clinical condition is not well-known [3, 6]. Thyroid nodules smaller than 1 cm in size are mostly nonpalpable. In the literature, the gold standard for the thyroid nodule(s) is thyroid USG; even in 1-2 mm sized nodules, thyroid USG is superior to CT and MRI for detecting thyroid nodule size and number. Therefore, thyroid USG should be performed in suspected cases [7].

The aim of USG guided FNAB is to detect malignant disease if it is present. Unfortunately, the rate of detecting malignant disease is approximately 50%. The main reason of low clinical value of FNAB in these cases is hypocellularity caused by dilution of cellular material with cystic fluid [3, 4, 6].

Papillary carcinoma is the most common type of thyroid malignant diseases, which is usually presented as a thyroid palpable mass [8]. On the other hand, cervical metastasis as an initial symptom in occult thyroid papillary carcinoma has been stated to occur approximately in one-fifth of all cases. Metastatic lymph nodes usually occur in the anterior and lateral side of the neck as solid masses [6, 9]. Although it is a rare phenomenon, as in our case, apparently benign cystic neck mass can be an initial symptom of an occult papillary thyroid microcarcinoma reported in no more than 50 cases in the literature (Table 1) [3].

RAI ablation is recommended for selected patients with 1–4 cm thyroid cancers confined to the thyroid gland, who have documented lymph node metastases or other higher risk features when the combination of age, tumor size, lymph node status, and individual histology predicts an intermediate to high risk of recurrence or death from thyroid cancer [7].

Seven et al. presented four cases with papillary occult thyroid microcarcinoma in which the initial presentation of cases was lateral cystic mass mimicking branchial cleft cyst, with a ranging age between 18 and 37 (mean: 29) [3]. In our case, the patient was 37-year-old male patient. Their all cases had only one microcarcinoma which were less than 9 mm in diameter in the thyroid gland, and also in our case the thyroid gland had 2 foci of papillary carcinoma with diameters of 4 mm and 6 mm, respectively. As a striking point, they also stated that they had no nodule on thyroid ultrasonography (USG) in one case, but histopathological examination of thyroidectomy specimen disclosed a unifocal 4 mm in diameter of papillary thyroid carcinoma. Therefore, even though there is no nodule on thyroid USG, thyroidectomy should be performed to rule out an occult primary tumor in the thyroid gland.

Gourin and Johnson presented 12 patients out of 121 cases whose initial diagnosis was lateral cervical cyst, but examination of specimens revealed squamous cell carcinoma metastasis in whom primary tumor was found only in 6 cases (50%) [4]. As an important point, all of the cases were over 40 years old. It seems that patient's age is an important mark regarding the cellular type of metastasis if it is present. Primary occult tumor is generally papillary thyroid carcinoma for patients under forty years of age, whereas it is generally squamous cell carcinoma from Waldeyer's ring or no focuses those for greater than 40 years old patients regarding with lateral cystic neck metastasis.

In conclusion, lateral cyst on the neck is generally a benign lesion, which is probably a branchial cyst. Nevertheless, it can be an initial presentation of cystic enlarged lymph node due to a papillary occult thyroid microcarcinoma metastasis that mimics a branchial cyst in young adults. Therefore, the physician has the first impression of branchial cleft cyst or cystic metastatic lesions from oropharyngeal or other primary sites, in even young patients. Further, FNA biopsy under US guidance may be taken for careful examination of all neck structures and lesions including the thyroid gland. This may lead to a correct initial diagnosis of the lesion.

## Figures and Tables

**Table 1:** The clinical features and presented cystic neck mass sizes of the cases are shown.

	Case	Age/sex	CNM (cm)	FNAB	T nodule(s) size (mm)	RIA
Seven et al. [3]	1	18/F	4 × 3	Positive	5	+
	2	26/M	5 × 4	NP	8	+
	3	37/F	3 × 3	Negative	No (4 mm PTC focus)	+
	4	34/M	3 × 2	Negative	3	+

Current case	5	37/M	6 × 4	Negative	4, 6, and 12	+

CNM: cystic neck mass, FNAB: fine needle aspiration biopsy, T: thyroid, RIA: radioactive iodine ablation, NP: not performed, and PTC: papillary thyroid carcinoma.
